# Obesity-induced blood-brain barrier dysfunction: phenotypes and mechanisms

**DOI:** 10.1186/s12974-024-03104-9

**Published:** 2024-04-27

**Authors:** Ziying Feng, Cheng Fang, Yinzhong Ma, Junlei Chang

**Affiliations:** 1 Key Laboratory of Biomedical Imaging Science, Shenzhen Institute of Advanced Technology, System of Chinese Academy of Sciences, Chinese Academy of Sciences, Shenzhen, Guangdong China; 2 University of Chinese Academy of Sciences, Beijing, China; 3 Institute of Biomedicine and Biotechnology, Shenzhen Institute of Advanced Technology, Chinese Academy of Sciences, Xueyuan Ave 1068, Nanshan, Shenzhen, 518055 Guangdong China

**Keywords:** Obesity, Blood-brain barrier, Neuroinflammation, Vascular permeability, Neurological disorders

## Abstract

Obesity, a burgeoning global health issue, is increasingly recognized for its detrimental effects on the central nervous system, particularly concerning the integrity of the blood-brain barrier (BBB). This manuscript delves into the intricate relationship between obesity and BBB dysfunction, elucidating the underlying phenotypes and molecular mechanisms. We commence with an overview of the BBB’s critical role in maintaining cerebral homeostasis and the pathological alterations induced by obesity. By employing a comprehensive literature review, we examine the structural and functional modifications of the BBB in the context of obesity, including increased permeability, altered transport mechanisms, and inflammatory responses. The manuscript highlights how obesity-induced systemic inflammation and metabolic dysregulation contribute to BBB disruption, thereby predisposing individuals to various neurological disorders. We further explore the potential pathways, such as oxidative stress and endothelial cell dysfunction, that mediate these changes. Our discussion culminates in the summary of current findings and the identification of knowledge gaps, paving the way for future research directions. This review underscores the significance of understanding BBB dysfunction in obesity, not only for its implications in neurodegenerative diseases but also for developing targeted therapeutic strategies to mitigate these effects.

## Introduction

Obesity, characterized by excessive fat accumulation, represents a burgeoning health issue of global populations. The World Health Organization reports an alarming rise in obesity rates worldwide, with an estimated 2.5 billion adults being overweight (body mass index, BMI > 25), of which approximately 890 million are obese (BMI > 30) as of 2022 [1]. This pervasive phenomenon profoundly impacts human health, inciting a multitude of disorders including cardiovascular disease, stroke, diabetes, and certain types of cancer.

Of particular interest, however, is the intricate relationship between obesity and neurological disorders, a link that has increasingly captivated scientific attention. Alzheimer’s disease [2–4] and stroke [5–7], for instance, are two representative neurological disorders that have been recurrently associated with obesity. Recent evidence points to obesity as a critical risk factor for mitochondrial dysfunction, as astrocytic fatty acid oxidation (FAO) and oxidative phosphorylation (OxPhos) are involved in these lipid-involving neurodegenerative disorders, implicating obesity’s role in influencing the disease onset and progression [8, 9]. This escalating prevalence of obesity and its potential implications for neurological disorders presents a grave concern for global health. It necessitates a comprehensive understanding of obesity’s detrimental consequences towards the central nervous system (CNS), specifically its effects on the cerebral vascular system.

A critical component of the cerebrovascular system is the blood-brain barrier (BBB), a critical function of brain blood microvessels that maintains CNS homeostasis. The BBB strictly regulates the exchange of substances between the blood and the brain. This dynamic barrier ensures the stability of brain microenvironment, protects against neurotoxins, and facilitates proper neuronal function [10, 11]. However, in pathological states such as obesity, this pivotal barrier can be compromised, leading to various neurological disorders. Understanding the intricate relationship between obesity and BBB dysfunction therefore becomes a matter of paramount importance.

We aim to delve into the complexities of this relationship, exploring the phenotypes and underlying mechanisms of BBB dysfunction in obesity. Through a comprehensive examination of the current literature, we provide an in-depth analysis of the potential changes in BBB structure and function induced by obesity. Additionally, we elucidate the mechanistic pathways that may be implicated in BBB dysfunction in obesity. This review ultimately seeks to underscore the significance of understanding BBB dysfunction in obesity and its implications for neurological health, with the goal of guiding future research and clinical practice.

## Blood-brain barrier: cellular and molecular components

**Fig. 1:**
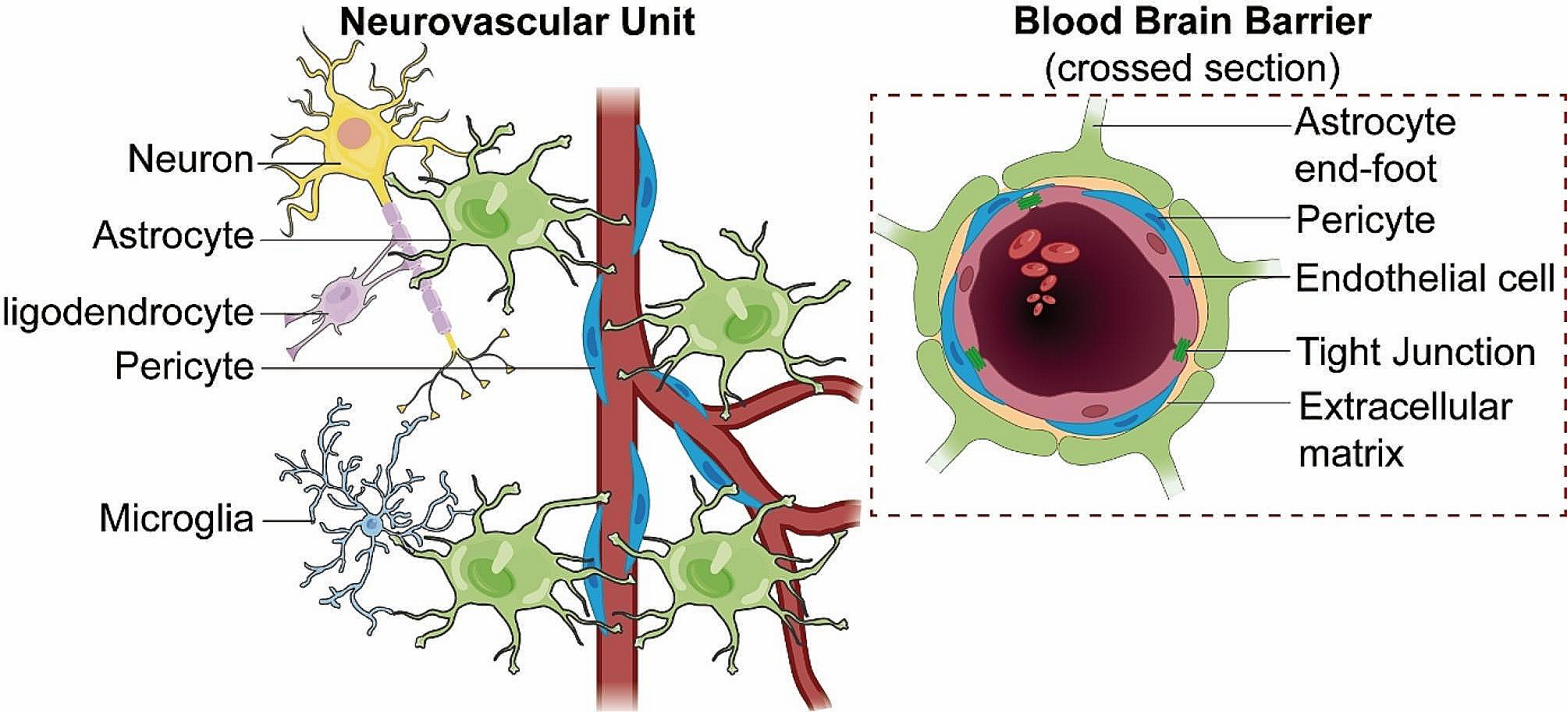
**BBB integrated within the NVU**. This diagram depicts the structural components of the blood-brain barrier (BBB) within the neurovascular unit (NVU), illustrating the tight junctions of endothelial cells, the supportive pericytes, and the encircling astrocytic end-feet. It highlights the BBB’s integral structural role in the NVU, which is essential for maintaining cerebral integrity and function

The blood-brain barrier (BBB) is a sophisticated and highly specialized biological construct, known for its selective permeability and protective role within the CNS. It owes its unique functionality to its intricately assembled cellular and molecular components, which work together to maintain CNS homeostasis. The structural and functional foundation of the BBB is the neurovascular unit (NVU) [12], a complex ensemble encompassing brain endothelial cells (BECs), pericytes, astrocytes, extracellular matrix (ECM), microglia, and neurons (Fig. 1).

### Endothelial cells

As the primary component of the BBB, brain endothelial cells (BECs), line the interior surfaces of the brain’s capillaries. These cells are distinctive in their unique morphology and possess attributes that separate them from peripheral ECs [13–15]. BECs are closely interconnected through tight junctions (TJs), which restrict the paracellular pathway and control the ionic balance across the barrier. This feature is instrumental in preventing the free passage of substances from the blood into the brain. BECs also exhibit limited pinocytic activity and lack fenestrations, thereby further restricting transcellular transport. In order to provide brain with abundant nutrients and a healthy environment, specific transporters which allows for the selective passage of molecules are expressed in BECs. Through analyzing single-cell RNA-sequencing data of mouse ECs from different organs provided by the *Tabula Muris* consortium, Paik et al. found that BECs possess not only its own unique transcriptomic identities, but also the most specialized differentially expressed genes (DEGs) profiles that expressed primarily solute carrier transporters [16]. For instance, glucose transporter protein-1(Glut-1) is responsible for transporting glucose, which being a crucial energy fuel, from blood to the brain [17]. On the other hand, P-glycoprotein (P-gp) acts as an efflux pump that removes harmful substances out of the brain to keep a safe environment [18].

Worth noticed, BECs can also transport molecules through low rates of transcytosis, a process that plays a vital role in endothelial transport [19, 20]. Transcytosis allows for selective or non-selective passage of molecules across the endothelial cells. In BECs, transcytosis can be divided into receptor-mediated (selective) transcytosis, which includes the transport of transferrin and insulin, and bulk-phase or fluid-phase (non-selective) transcytosis, typically exemplified by the transport of albumin [21]. Notably, while native albumin crosses the BBB via non-selective bulk-phase transcytosis, cationic albumin is transported more efficiently as it engages in adsorptive transcytosis, highlighting the distinct pathways for different forms of albumin. A key regulator in this process is Major Facilitator Superfamily Domain Containing 2 A (Mfsd2a), which specifically inhibits bulk-phase transcytosis in BECs [22]. Mfsd2a modifies the lipid composition of the cell membrane, thereby limiting the formation of caveolae vesicles and reducing caveolae-mediated transcytosis. This action helps maintain the selective permeability and overall integrity of the BBB. Contrary to inhibiting all forms of transcytosis, Mfsd2a’s role is particularly pivotal in restraining non-selective transcytosis or bulk-phase transcytosis. Research indicates that overexpression of Mfsd2a in CNS endothelial cells leads to a decreased number of transcytotic vesicles, which in turn reduces hematoma levels and alleviates BBB injury in mouse models of intracerebral hemorrhage (ICH) [23]. This highlights the potential therapeutic value of Mfsd2a in mitigating BBB disruption by specifically targeting non-selective transcytosis mechanisms post-injury.

In comparison to endothelial cells found elsewhere in the body, brain endothelial cells (BECs) demonstrate a unique profile of leukocyte adhesion molecule (LAM) expression. This profile contributes to the CNS’s immune privilege by limiting the extravasation of leukocytes under normal conditions. While LAMs are integral to leukocyte adhesion and migration — key steps in the immune response — their regulated expression in BECs ensures a controlled interaction with leukocytes, thereby maintaining CNS homeostasis [24]. Contrary to the constant expression in peripheral tissues, the presence and activity of LAMs in the BBB vary contextually. Notably, BECs typically lack selectins in their Weibel-Palade bodies, which reduces their ability for rapid leukocyte recruitment compared to peripheral endothelial cells. This does not equate to a complete suppression of immune response but represents a finely tuned regulation ensuring selective leukocyte passage without compromising BBB integrity [25]. During certain pathological conditions, leukocytes such as neutrophils and T cells may interact with the BBB in ways that can affect its permeability, through the release of cytokines, reactive oxygen species, and other mediators. However, these interactions do not universally result in BBB disruption and can occur in the absence of significant leakage, depending on the specific disease context and the nature of leukocyte engagement. In the context of neuroinflammatory diseases, such as multiple sclerosis (MS), alterations in the expression and functionality of LAMs have been observed. These changes can facilitate increased adhesion of leukocytes to BECs, potentially leading to BBB disruption under these pathological states [26]. However, this process reflects a departure from the normative regulatory mechanisms in place under healthy conditions and underscores the complex role of LAMs in both maintaining CNS immunity and contributing to neuroinflammation when dysregulated.

### Pericytes

Pericytes is a kind of multi-functional cells embedded within the walls of capillaries throughout the body, including the brain, and play a crucial role in maintaining the integrity of the BBB. They were first identified in the 1870s, and more recently, numerous vascular functions of pericytes have been identified. These include regulation of cerebral blood flow, maintenance of the BBB integrity, and control of vascular development and angiogenesis [27–29]. In addition to these roles, pericytes have been found to play an active role during neuroinflammation in the adult brain [28, 30, 31]. They can respond differentially, depending on the degree of inflammation, by secreting a set of neurotrophic factors to promote cell survival and regeneration, or by potentiating inflammation through the release of inflammatory mediators (e.g., cytokines and chemokines), and the overexpression of pattern recognition receptors [32]. In neuroinflammatory conditions like multiple sclerosis, pericytes undergo morphological changes, elongating their processes within inflamed perivascular cuffs [33]. Exposure to cytokines and extracellular matrix proteins like chondroitin sulfate proteoglycans enhances pericyte secretion of chemokines and promotes macrophage migration [33]. This implicates pericytes in propagating neuroinflammation through immune cell recruitment. However, pericytes also have neuroprotective capacities dependent on the degree of inflammation [34]. Through release of neurotrophic factors like BDNF, pericytes can promote neuronal survival and regeneration [35]. Their expression of cell adhesion molecules likewise facilitates interactions with endothelial cells and astrocytes to maintain cerebrovascular stability [36]. The dual functionality of pericytes is highlighted by their differential secretome profiles in response to IL-1β [37]. While pericytes secrete certain pro-inflammatory genes like CCL2 [38], they also show expression of vascular-stabilized mediators like TIMP3 [34, 39]. This nuanced, context-dependent pericyte reactivity fine-tunes neuroinflammatory responses.

Pericyte dysfunction is increasingly recognized as a contributor to the progression of vascular diseases such as stroke and neurodegenerative diseases [40, 41]. The therapeutic potential of pericytes to repair cerebral blood vessels and promote angiogenesis due to their ability to possess stem cell-like properties has recently been brought to light. In the context of cerebral blood vessels repair, pericytes can migrate to the site of injury and differentiate into cells that are needed for repair [42, 43]. As for promoting angiogenesis, pericytes play a vital role in the formation of new blood vessels from pre-existing ones, a process known as angiogenesis [44]. They stabilize the newly formed endothelial tubes, modulate blood flow and vascular permeability, and regulate endothelial proliferation, differentiation, migration and survival. However, research has shown that pericytes and endothelial cells have overlapping but distinct secretome profiles in response to IL-1β [45]. This indicates that these two cell types may respond differently to inflammatory stimuli, which could have implications for understanding how inflammation affects the cerebrovasculature.

In conclusion, pericytes play a critical role in maintaining BBB integrity by controlling various processes. Understanding these mechanisms could provide valuable insights into BBB function and CNS immunity. Future research directions could include exploring the role of other proteins involved in these processes, further investigating the function of pericytes in disease states, and studying how changes in pericyte function could impact BBB integrity and CNS health.

### Astrocytes

Astrocytes, another critical component of the NVU, also play a crucial role in BBB maintenance. Their end-foot processes enwrap the brain capillaries and provide physical support to the BBB. Through their extensive contact with endothelial cells, astrocytes are vital in the formation and maintenance of the BBB. More specifically, astrocytes can secrete Sonic hedgehog (Shh), thereby stimulating the expression of TJs proteins and junctional adhesion molecule-A (JAM-A) while promoting immune quiescence of BBB by decreasing the expression of chemokines and LAMs [46]. The Hedgehog (Hh) pathway is involved in embryonic morphogenesis, neuronal guidance, and angiogenesis [47]. In adult tissues, it participates in vascular proliferation, differentiation, and tissue repair. The Hh pathway provides a barrier-promoting effect and an endogenous anti-inflammatory balance to CNS-directed immune attacks [48]. In terms of cerebrovascular accidents, astrocytes have been found to play a crucial role in maintaining blood-brain barrier function [49]. They support neurons and other glia, and react to changes in both the local and external environment. Beyond these homeostatic functions, astrocytes can respond to several stimuli and subsequently display profound genetic, morphological, and functional changes in a process termed reactive astrogliosis.

In addition to their role in maintaining BBB integrity, astrocytes also play a crucial role in controlling water homeostasis in the brain. Aquaporins (AQPs) are transmembrane proteins responsible for fast water movement across cell membranes, including those of astrocytes [50]. Various subtypes of AQPs (AQP1, AQP3, AQP4, AQP5, AQP8 and AQP9) have been reported to be expressed in astrocytes. The expressions and subcellular localizations of AQPs in astrocytes are highly correlated with both their physiological and pathophysiological functions [51, 52].

### Basement membrane

The basement membrane (BM) was secreted and maintained by BECs, pericytes and astrocytes. The BM is composed of various proteins, including Laminins, Collagen-IV, Perlecan, and Fibronectin [53, 54]. These components interact with each other and with other cells at the BBB. For example, Laminins provide structural support to the BBB and are involved in processes such as cell adhesion, differentiation, migration, and apoptosis, while Collagen-IV provides mechanical strength to the BBB and contributes to its stability [53, 54]. Any alterations in these components can lead to BBB dysfunction, which is associated with various neurological disorders. For instance, in conditions such as stroke, the BBB undergoes significant changes, including alterations in the expression of integrins and degradation of surrounding ECM, which indirectly affect the vascular barrier function [55]. In an animal model of autoimmunity, considerable information has been revealed about the function of different BMs in maintaining BBB integrity and causing neuroinflammation [56–58].

ECM receptors, such as integrins and dystroglycan, are also expressed at the brain microvasculature and mediate the connections between cellular and matrix components in physiology and disease [59–61]. These proteins and receptors elicit diverse molecular signals that allow cell adaptation to environmental changes and regulate growth and cell motility [62].

### Microglia and neurons

Finally, microglia and neurons, while not directly forming the BBB, significantly contribute to its function and maintenance. Microglia, the resident immune cells of the brain, play a crucial role in maintaining the integrity of the BBB. Following ischemic stroke, microglia interact with endothelial cells in a paracrine manner to promote angiogenesis and barrier repair [63]. However, this reparative crosstalk is impeded in aging, implicating declined microglial function in age-related BBB disruption [64]. Accordingly, experimental depletion of microglia exacerbates injury and edema in aged mice subjected to ischemic stroke, further confirming microglia’s protective influence [65]. Beyond strokes, microglia also maintain BBB stability in neuroinflammatory conditions by modulating astrocyte reactivity and phagocytosing synapses [66]. In a model of multiple sclerosis, microglia limited astrocyte activation and protected against BBB leakage, stressing their homeostatic role [67]. During reactive gliosis induced by stroke, microglia and macrophages preferentially eliminated excitatory synapses while astrocytes cleared inhibitory synapses, preventing excessive imbalance of excitatory/inhibitory tone [67].These findings underscore diverse mechanisms whereby microglia maintain cerebrovascular equilibrium, ranging from paracrine support of angiogenesis to controlled synapse engulfment. Their protective influence likely declines with aging, permitting BBB hyperpermeability. Boosting microglial function represents a promising strategy to strengthen BBB integrity in neurological disorders.

Neurons also play a significant role in BBB’s function and maintenance. Neurons communicate with other components of the neurovascular unit (NVU), such as endothelial cells, pericytes, and astrocytes, to regulate BBB properties. This communication is also critical for maintaining the CNS homeostasis and for responding to changes in neural activity. In the early postnatal mouse barrel cortex, it was demonstrated that manipulations of sensory inputs resulted in vascular structural changes [68]. Specifically, this study showed that local sensory-related neural activity promoted the formation of cerebrovascular networks.

Neurovascular coupling refers to the relationship between local neural activity and subsequent changes in cerebral blood flow (CBF) [69]. The magnitude and spatial location of blood flow changes are tightly linked to changes in neural activity through a complex sequence of coordinated events involving neurons, glia, and vascular cells. This mechanism is crucial as it matches the high energy demand of the brain with a supply of energy substrates from the blood. Evoked the neural activity by high-intensity visual stimulation could drive macroscopic cerebrospinal fluid (CSF) flow in the human brain [70]. The timing and amplitude of CSF flow were matched to the visually evoked hemodynamic responses, suggesting neural activity can modulate CSF flow via neurovascular coupling.

In addition to their role in neurovascular coupling, neurons also contribute to BBB function through some transporters. It was found that neuronal activity regulates BBB efflux transporter expression and function [71], which is critical for excluding many small lipophilic molecules from the brain parenchyma. These findings suggest that sensory-related neural activity can influence both vascular structure and BBB function, which could have significant implications for understanding neuro-vascular interaction. An understanding of these intricate structures and their functions is pivotal in comprehending how pathological states, such as obesity, could affect the BBB and ultimately lead to neurological disorders.

## Obesity-induced BBB leakage: phenotypes and mechanisms

The impact of obesity on BBB integrity has been an area of increasing scientific scrutiny, given the relationship between obesity and various neurological disorders. The disruption of BBB integrity or non-specific leakage of the BBB, as it is commonly referred to, is a recurrent phenotype observed in obesity, especially in the context of high-fat diet (HFD) intake with a wide duration from 8 to 36 w [72–75]. This section seeks to elucidate the molecular mechanisms and phenotypic changes underlying obesity-induced BBB leakage and associated BBB markers of this pathological state.

### Changes in tight junctions

A key factor implicated in obesity-induced BBB leakage is the dysregulation of tight junction proteins (TJs). TJs, as earlier stated, are critical in maintaining the restrictive properties of the BBB. However, obesity, particularly under conditions of HFD, can alter the expression and function of these proteins. For instance, Ouyang et al. observed alterations in the expression levels of ZO-1 in microvessels from obese mice modeling by 8 weeks (w) of HFD [76]. In line with this observation, HFD significantly decreased the protein levels of ZO-1, Claudin-5 and Occludin along with the leakage of brain microvessles after 8 weeks (w) of HFD [77]. In adult rats, 90 days of high-energy diet (high in saturated fat and glucose) consumption decreased mRNA expression of TJs, particularly Claudin-5 and − 12, in the choroid plexus and the BBB. Consequently, an increased blood-to-brain permeability of sodium fluorescein was observed in the hippocampus [78]. This underscores the potential for obesity-induced modifications to BBB structure and function.

The adenosine receptor 2a (Adora2a) is increasingly recognized for its significant role in the modulation of neurovascular and neuroinflammatory responses. Activation of Adora2a receptors has been linked to heightened inflammatory responses, contributing to the disruption of the blood-brain barrier (BBB) and subsequent neuronal damage, conditions often exacerbated by obesity and metabolic syndrome. In a rodent model of diet-induced insulin resistance by 16 w of HFD, it was found that chronic activation of Adora2a eroded TJs between BECs, as evidenced by diminished Occludin and Claudin-5 in hippocampal lysates. Considering the detrimental effects associated with Adora2a activation on BBB integrity, antagonism of this receptor presents a promising therapeutic strategy. By inhibiting Adora2a, it is possible to mitigate the receptor-mediated exacerbation of inflammatory processes within the CNS, thereby preserving BBB function and reducing neuroinflammatory sequelae. This premise is supported by several studies demonstrating that Adora2a antagonists can effectively reduce BBB permeability and alleviate inflammatory damage in various neuroinflammatory and neurovascular disorders [79–81].

In addition, it is noteworthy that obesity not only affects one’s own BBB function, but also has an impact on its offspring. A recent study showed that maternal obesity during pregnancy could impaired BBB formation of the fetal, leading to changes in TJ components of the arcuate nucleus region in offspring’s brain, thereby significantly increasing BBB permeability [82]. This dysregulation of TJs compromises the integrity of the BBB, increasing its permeability and enabling the passage of potentially harmful substances from the blood into the CNS. Interestingly, recent studies have also linked prolonged HFD intake for 32 w to anxiety-like and depression-like behaviors in mice [83]. It was found that 24 weeks of HFD consumption induced neurobehavioral deterioration, including increased anxiety-like and depression-like behavior. These behavioral changes were associated with impaired gut microbiota homeostasis and inflammation. Long term HFD may induce certain behavioral phenotypes related to neurological disorders through the gut-brain axis [83]. In line with this, treatment with the anti-inflammatory molecule palmitoylethanolamide was found to reduce anxiety-like behavior in obese mice modeling by 19 w of HFD, along with dampening systemic and central inflammation [84]. Taken together, these studies suggest prolonged HFD may not only directly disrupt TJs and BBB integrity through inflammatory and other mechanisms, but also trigger neurobehavioral changes that could secondarily impact BBB function.

### Changes in fenestration

As mentioned above, BECs are characterized by a lack of fenestrations, a characteristic that contributes to the high selectivity and restrictive nature of the BBB. Stan et al. identified PV-1 (also known as PLVAP, plasmalemma vesicle-associated protein; or MEGA-32 antigen) as a component of fenestral diaphragms in endothelial cells [85]. As PV-1 comprises these structures, its regulation could influence fenestration numbers. While PLVAP expressed on fenestrated endothelia and associated with the formation of diaphragms in vesicular structures, it does not serve as a marker for fenestrations within the BBB context. Instead, increases in PLVAP expression may reflect alterations in the molecular composition associated with transcellular pathways rather than the formation of true fenestrae. In a single-cell profiling study, analysis of BECs revealed that among eight major clusters, fenestrated BECs in areas such as the choroid plexus showed the most unique obesity-induced DEGs, where fenestrated endothelia are typical, rather than suggesting the emergence of fenestrations within the BBB due to obesity modeling by 12 w of HFD [86]. In line with this, Previous study have demonstrated that HFD intake can lead to an increase in endothelial fenestration in the BBB, such as the observed changes in the offspring of gestational obesity in mice [82]. Worth noticed, these findings, particularly those related to increased permeability in regions like the arcuate nucleus, may reflect localized alterations in BBB properties rather than systemic induction of fenestrations akin to those in inherently fenestrated structures like the choroid plexus.

Therefore, while the effects of HFD on endothelial cell biology are undeniable, the notion that these effects lead to the formation of fenestrations within the typical BBB structure remains debatable. This perspective allows for the possibility that PLVAP-regulated alterations may occur regionally within specific areas of the brain under certain conditions, rather than implying a global change across the entire barrier. The DEGs identified in obesity models, particularly those affecting endothelial cells of the BBB, should be interpreted with an understanding that obesity-induced stress may lead to changes in molecular signaling and barrier properties without necessitating the creation of actual fenestral openings.

### Changes in matrix metalloproteinases

**Fig. 2:**
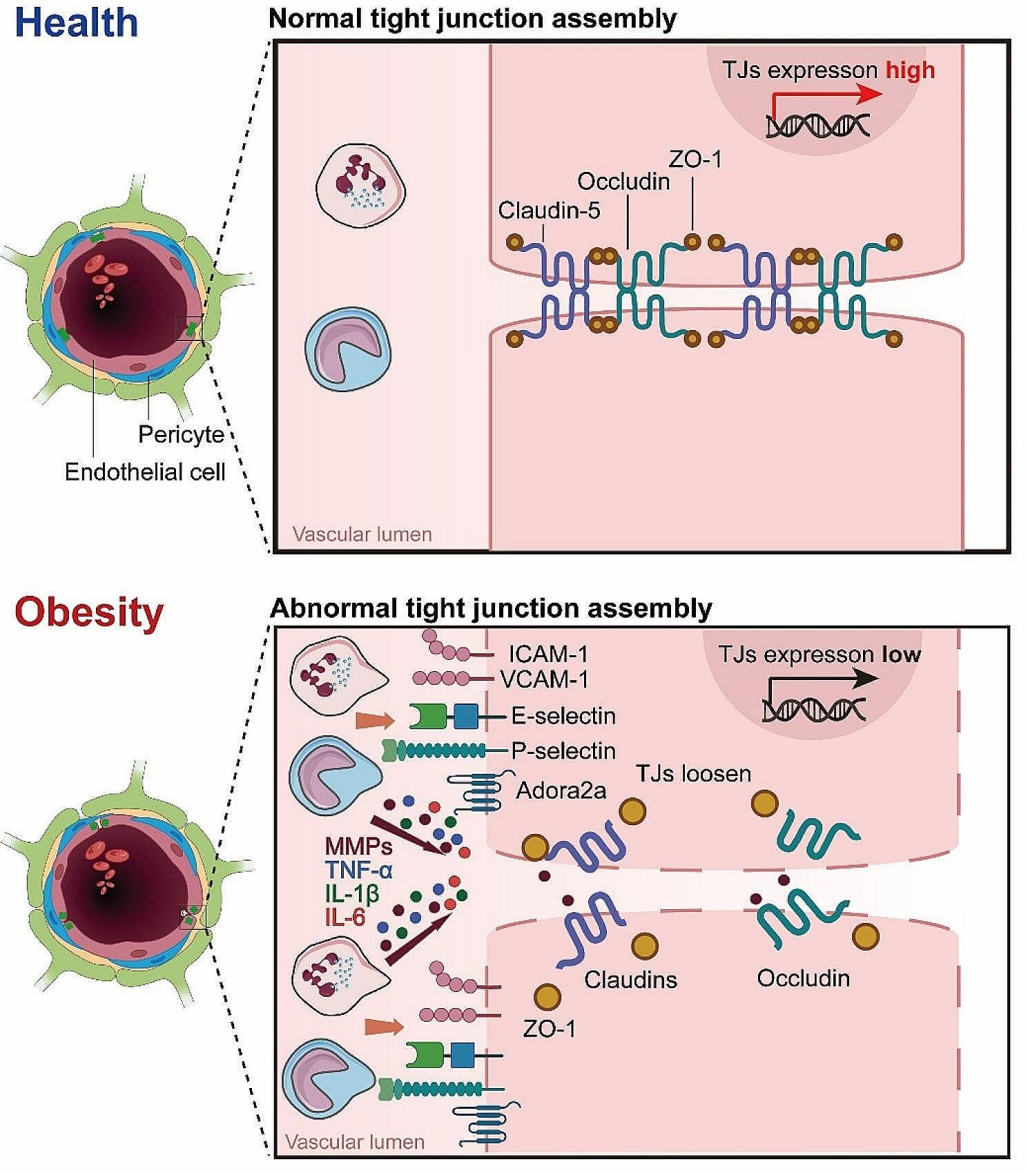
**Mechanisms of Obesity-Induced BBB Leakage.** Illustration of the complex alterations in the BBB induced by obesity, highlighting the loosening of endothelial cell junctions, increased permeability, and the unusual occurrence of fenestrations. These structural changes are further exacerbated by the activity of MMPs, which degrade extracellular matrix components, facilitating the infiltration of peripheral immune cells, such as neutrophils. This cascade of events underscores the multifaceted and interconnected impact of obesity on BBB integrity and the resulting immune response within the cerebral vasculature

Matrix metalloproteinases (MMPs) are a family of endopeptidases that function to degrade and remodel the extracellular matrix (ECM). MMPs are secreted by various cell types including epithelial cells, fibroblasts, and inflammatory cells, playing important roles in physiological processes as well as disease states characterized by tissue damage and inflammation. However, excessive MMPs can lead to pathological ECM degradation and impairment of tissue structure and function, as in the case of blood-brain barrier (BBB) disruption by MMP-2 and MMP-9 [87–89]. Obesity has been linked to increased expression and activity of certain MMPs, which can impair BBB integrity. For example, plasma MMP-9 levels were found to be elevated in obese subjects and decreased with anti-diabetic treatment [90, 91]. Cotemporally, lipocalin-2 was also increased in adipose tissue of obese individuals and correlated with MMP-2 and MMP-9 activity [92]. MMP-8 levels were similarly increased in obesity and associated with insulin resistance [93]. Additional studies have assessed levels of MMPs like MMP-2 and MMP-9 along with their inhibitors TIMPs in obese children [94]. Together, these findings suggest obesity creates a pro-inflammatory state characterized by upregulation of MMPs like MMP-8 and MMP-9, potentially driven by increases in mediators like lipocalin-2. By degrading ECM proteins, these MMPs can impair BBB structural integrity. A general description of obesity-induced BBB leakage was shown in Fig. 2.

## Obesity-induced BBB transport dysfunction: phenotypes and mechanisms

In addition to disrupting the BBB integrity, obesity is associated with alterations in the BBB’s transport functions. These changes not only affect the nutrient supply to the brain but can also influence the entry and clearance of toxins, signaling molecules, and therapeutics. The transport across the BBB is highly regulated, with specific receptors and transporters expressed in BECs mediating the process. However, obesity can induce significant changes in these receptors and transporters, leading to BBB transport dysfunction.

### Glucose transport-1 (Glut-1)

One of the key alterations in BBB transport function under obesity conditions is the dysregulation of glucose transport. The primary transporter responsible for glucose transport across the BBB is the glucose transporter 1 (Glut-1), which ensures the supply of glucose from the blood to the brain. However, in the state of obesity, particularly in HFD-induced obesity, the expression and function of Glut-1 have been found to be altered. Specifically, studies in mouse models have demonstrated that acute high-fat feeding for 2–3 days suppressed Glut-1 expression and glucose uptake in the brain [95, 96]. Similarly, the mouse insulin resistance model showed that when 2 weeks of HFD significantly downregulated the expression of Glut-1 in the brain, 10 weeks of HFD normalized the expression of Glut-1, suggesting the duration of insulin resistance may influenced the regulation of Glut-1 [97]. In humans, genetic factors were found to impact Glut-1 levels, which in turn modulates cognitive effects of high-fat intake [98].

Interestingly, upon more prolonged high-fat feeding for 10 weeks, Glut-1 expression was restored, which was related to the initiation of compensatory mechanisms of vascular endothelial growth factor (VEGF), a key regulator of angiogenesis and vascular function [95]. Recent studies have illuminated the role of VEGF in respond to metabolic stresses and hypoxic conditions often prevalent in obesity. This response includes the potential to upregulate Glut-1 expression, ostensibly to compensate for altered metabolic demands and ensure sufficient glucose transport across the BBB. However, the interplay between VEGF and Glut-1 in the setting of obesity is multifaceted. While moderate increases in VEGF can be beneficial, aiding in the restoration of Glut-1 levels and maintaining cerebral glucose metabolism, chronic elevations—common in prolonged obesity—may lead to adverse effects [95, 96]. Excessive VEGF can contribute to vascular abnormalities and exacerbate BBB disruption, complicating the metabolic landscape of the CNS. Therefore, understanding the dynamic between VEGF-induced Glut-1 modulation and obesity provides insight into the broader implications of metabolic syndrome on BBB health and brain metabolism. This section explores how the nuanced changes in VEGF and Glut-1 expression influenced by obesity underscore the complexity of maintaining BBB integrity and highlight the need for targeted therapeutic strategies to address these metabolic challenges.

Taken together, while short-term high-fat intake appears to impair Glut-1 function and glucose transport at the BBB, compensation may occur with prolonged obesity to normalize Glut-1 expression. However, the brain is the most energy-consuming organ, requiring constant high energy to maintain its function, thereby the temporary disruption in glucose delivery to the brain may be sufficient to impact neuronal health and cognition. Further research into the kinetics of Glut-1 regulation in response to high-fat feeding could delineate the timeframe of BBB transport deficits. This may provide insights into critical windows where impaired brain glucose uptake contributes to neurological disorders associated with obesity.

### Insulin

Alterations in insulin transport have also been observed in obesity, which can profoundly impact neuronal function. Insulin enters the brain via saturation transport and binds to insulin receptors (IR) on BECs, triggering receptor autophosphorylation and downstream signaling cascades involved in glucose uptake, metabolism, neuronal plasticity and survival [99]. However, studies in obese animal models and human subjects indicate that obesity and HFD feeding can impair insulin transport across the BBB. In preclinical studies using mouse and canine models, HFD feeding for 7 w reduced transport of intravenously injected insulin from the circulation into the brain parenchyma [100, 101]. This reduction is associated with central insulin resistance, as evidenced by impaired insulin receptor signaling cascades in the brain from mice subjected to 4 w of HFD [102]. In human clinical studies, obese subjects were found to have lower cerebrospinal fluid (CSF) insulin levels and attenuated CSF insulin increases after systemic insulin infusion compared to healthy controls [103]. Together, these animal and human studies demonstrate that obesity disrupts brain endothelial insulin transport, reducing insulin delivery to the CNS. This transport dysfunction contributes to central insulin resistance, a phenomenon often linked with cognitive dysfunction and neurological disorders [104]. Consistence with this, brain insulin resistance has been recognized as an early characteristic of Alzheimer’s disease [105]. Elucidating the mechanisms by which high-fat feeding alters insulin receptor expression, trafficking and downstream signaling at the BBB will be critical for developing therapeutic strategies to overcome CNS insulin resistance.

### Leptin

In addition to insulin, the transport of the hormone leptin across the BBB is also altered in obesity. Leptin is also transported into the brain via a saturable transport system and binds to leptin receptors on neurons involved in regulating food intake and energy expenditure. However, multiple studies indicate HFD-induced obesity impairs leptin transport across the BBB. For instance, in obese individuals, the transport level of leptin to the brain is downregulated, as evidenced by a significantly lower ratio of the leptin cerebrospinal fluid (CSF)/serum compared to the healthy lean individuals [106, 107]. In obese animal models including rodents subjected to 10 w of HFD and sheep subjected to 40 w of HFD, there is a significant decrease in the rate of leptin transport from blood to brain compared to lean controls [108–110]. Obesity can inhibit the transport of leptin across the BBB, making it impossible for the brain to receive the “satiety signal” emitted by leptin, leading to overeating and worsening of obesity, which may lead to a series of metabolic diseases. Of notice, with the development of obesity, obese mice modeling by 56 days of HFD respond to leptin for central administration (intracerebroventricularly) rather than peripheral administration (intraperitoneally or subcutaneously) [111, 112]. This suggests that. the impairment in leptin transport does not appear to be due to altered leptin receptor expression at the BBB. Rather, transport may be inhibited due to saturation of the carrier system and interactions with other circulating factors, such as the high-level triglycerides [113–115]. Overall, these findings indicate obese states inhibit leptin’s ability to enter the CNS and bind neuronal targets, despite normal BBB leptin receptor levels. Overcoming the transport block could potentiate leptin’s effects on appetite and weight regulation.

### P-glycoprotein (P-gp)

Obesity also affects the function of efflux transporters at the BBB, such as the P-glycoprotein (P-gp). P-gp is an ATP-dependent transporter that functions to pump foreign substances and metabolites out of the brain back into the bloodstream. This helps protect the brain from accumulation of potentially toxic compounds. P-gp is encoded by the ABCB1 gene. A human study found a negative correlation between BMI values and the expression levels of *ABCB1* in the brain, suggesting P-gp levels are reduced in obesity [116]. While the mechanisms linking obesity to P-gp regulation require further elucidation, systemic inflammation appears to play a role. In obese pregnant mice, placental P-gp expression was decreased in tandem with increases in inflammatory cytokines like tumor necrosis factor-alpha (TNF-α), interleukin-1 beta (IL-1β), and interleukin-6 (IL-6) [117]. Changes in P-gp likely impair efflux of substrates from the brain back into circulation. Overall, although there is limited research on obesity and P-gp, current evidence indicates obesity can suppress P-gp expression and function at the BBB, at least in the obese human. This impairment in a key transporter for xenobiotic clearance may enable accumulation of toxins and drugs in the brain.

### L-type amino acid transporter-1 (LAT1)

**Fig. 3:**
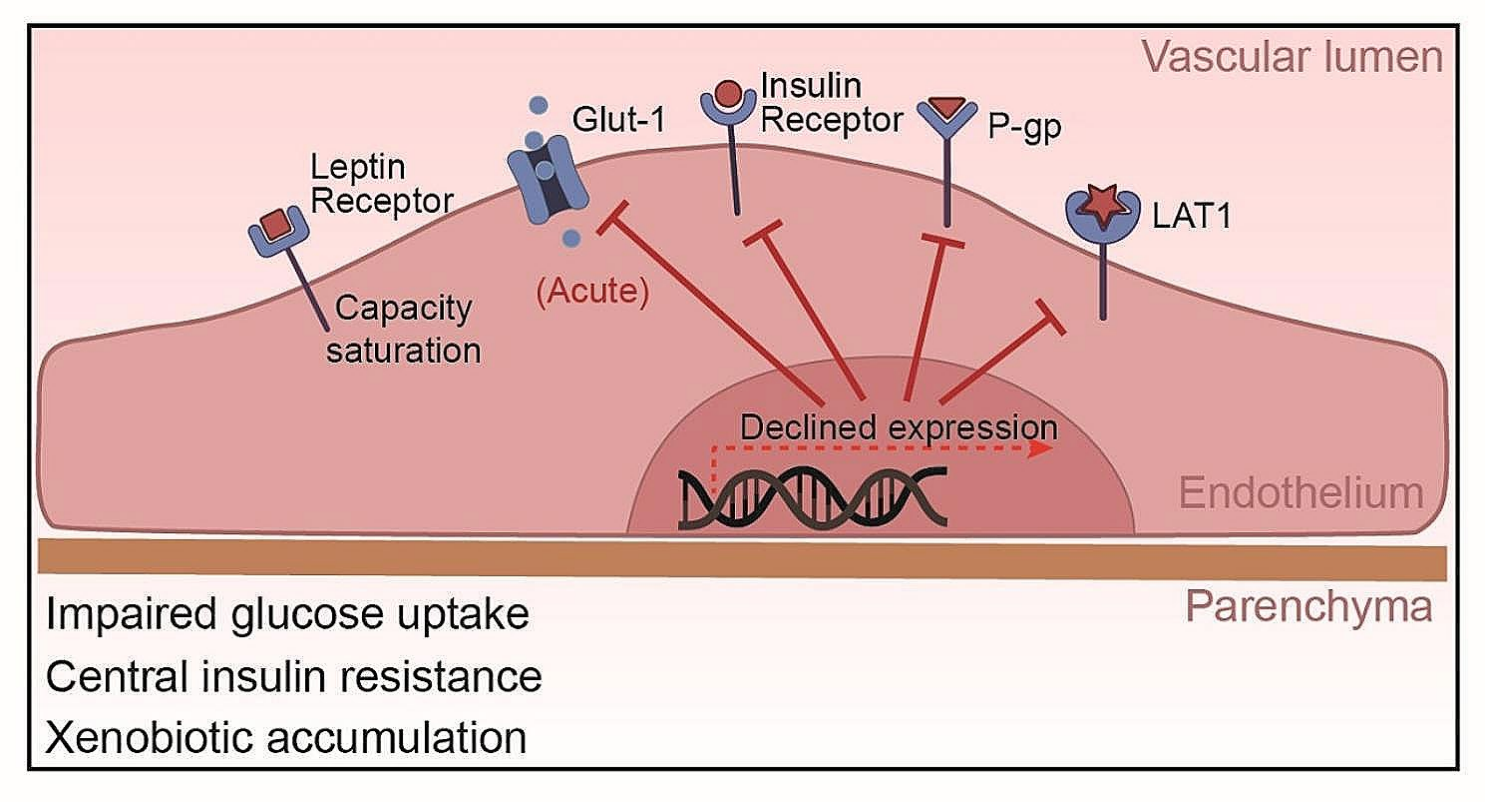
**Obesity-Induced Alterations in BBB Transporters.** This figure demonstrates the effects of obesity on BBB transporters: Glut-1 function is initially impaired by high-fat intake but may be normalized with prolonged obesity, despite potential impacts on neuronal health and cognition. Brain endothelial insulin transport is disrupted, contributing to central insulin resistance, and often associated with cognitive dysfunction and neurological disorders. Leptin transport impairment is linked to carrier system saturation, independent of leptin receptor expression changes. Obesity suppresses P-glycoprotein expression and function, potentially increasing brain accumulation of toxins and drugs. LAT1 expression and amino acid uptake are reduced in the hypothalamus of obese, diabetic mice, reflecting impaired amino acid transporter function at the BBB.

Amino acid transporters are also affected by obesity. The system L amino acid transporter 1 (LAT1) expressed at the BBB is responsible for transport of large neutral amino acids like leucine into the brain. LAT1 plays a key role in regulating mTORC1 signaling, which controls processes like protein synthesis and autophagy [118]. Recent studies have found that LAT1 function is altered in obesity models. Mice lacking neuronal LAT1 develop obesity phenotypes including increased adiposity [119]. LAT1 expression and amino acid uptake are reduced in the hypothalamus of obese, diabetic mice [120]. In humans, lower expression of the related transporter SLC7A10/ASC-1 in adipose tissue is associated with increased visceral fat, insulin resistance, and adipocyte hypertrophy [121]. Together, these findings indicate obesity impairs the function of multiple amino acid transporters at the BBB and periphery. This likely dysregulates mTORC1 signaling and other nutrient-sensing pathways, contributing to metabolic dysfunction. The alterations of transporters expressed in endothelial cells was summarized in Fig. 3.

### Evidence of BBB disruption in obesity and type 2 diabetes

Recent evidence has increasingly indicated that metabolic disorders such as obesity and Type 2 Diabetes (T2D) are closely linked to the disruption of the BBB. This section aims to elucidate the relationship between these conditions and BBB integrity, underpinned by recent scientific findings.

BBB Permeability in Type 2 Diabetes: Among the most compelling evidence in this domain is the study conducted by Starr and colleagues [122], which has demonstrated a significant increase in BBB permeability to gadolinium in patients with well-controlled T2D. This landmark study utilized advanced magnetic resonance imaging techniques to compare BBB integrity between diabetic patients and healthy individuals. The results indicated a pronounced increase in BBB permeability, notably in the basal ganglia region, suggesting a direct impact of T2D on neurovascular integrity. These findings are critical as they highlight the potential for well-controlled T2D to contribute to BBB dysfunction independently of other comorbidities often associated with metabolic syndrome.

The link between obesity and BBB disruption has been further substantiated by studies investigating the levels of specific BBB markers in obese individuals. A longitudinal perspective explores how factors related to adiposity in mid-life can have long-lasting effects on BBB integrity. The study highlights the enduring impact of obesity on neurovascular health, suggesting that the consequences of increased body weight extend far beyond the immediate metabolic disturbances commonly associated with obesity [123]. Another study focused on Adipsin, a complement system component known to be influenced by adiposity levels, and studied within the cerebrospinal fluid, providing insights into the biochemical pathways through which obesity might mediate changes in BBB integrity. This research suggests a direct link between metabolic health markers and the biochemical status of the BBB, illustrating the intricate connection between systemic metabolic health and neurovascular function [124]. These studies have shown elevated levels of certain markers, indicating compromised BBB integrity in the context of excessive body weight and associated metabolic derangements. The disruption is believed to be multifactorial, involving mechanisms such as increased systemic inflammation, altered lipid metabolism, and hypertension, all of which are prevalent in obesity and can adversely affect the endothelial cells constituting the BBB.

The mechanistic pathways through which obesity and T2D exacerbate BBB disruption are complex and multifaceted. Inflammatory cytokines, often elevated in obesity and T2D, are known to compromise BBB integrity by altering tight junction protein expression and endothelial cell function. Additionally, the hyperglycemic environment in T2D can induce oxidative stress and microvascular complications, further impairing BBB function. These alterations not only have direct implications for neurovascular health but also predispose individuals to a range of neurological disorders. Takechi et al. [125] shows that BBB dysfunction precedes cognitive decline and neurodegeneration in a diabetic insulin-resistant mouse modeled by 24 w of high fat and fructose fed, which may imply a causal link. Although this study is more centered around diabetes, the intersection with obesity (through insulin resistance) makes it pertinent. The above findings emphasizing the need for comprehensive management of these metabolic conditions to maintain BBB integrity and overall brain health.

In summary, the accumulating evidence underscores a significant association between obesity, T2D, and BBB disruption. This relationship highlights the importance of managing these metabolic disorders not only for cardiovascular health but also for maintaining the integrity of the neurovascular unit. As research in this field continues to evolve, understanding the specific pathways and impacts will be crucial for developing targeted interventions aimed at preserving BBB integrity in the face of growing obesity and T2D prevalence.

## Obesity-induced neuroinflammation: phenotypes and mechanisms

Neuroinflammation, characterized by the activation of resident brain cells (microglia and astrocytes) and infiltration of peripheral immune cells, has been widely recognized as a critical pathological feature of obesity. The increased BBB permeability induced by obesity, as discussed earlier, not only allows harmful substances to penetrate into the CNS but also paves the way for peripheral immune cells to infiltrate the brain. Once in the CNS, these immune cells can instigate an inflammatory response, contributing significantly to obesity-induced neuroinflammation.

### Microglia

Microglia, the primary immune cells of the CNS, show enhanced activation in obesity. Once activated, microglia release a plethora of pro-inflammatory factors, including cytokines like TNF-α, IL-1β, and IL-6, thereby promoting a pro-inflammatory environment within the CNS [84, 126–128]. In the research employed bone marrow chimerism mouse subjected to 15 or 30 w of HFD, it was also found that the aggregated inflammatory monocytes/macrophages located in the parenchyma and expressed the microglial marker Iba1 [129]. Notably, while microglia often exhibit a pro-inflammatory phenotype in the early stages of HFD-induced obesity, their activation state appears to change over time with prolonged exposure. As the study by Baufeld et al. [130] demonstrated, the initial microglial reaction in the hypothalamus of mice with 3 days of HFD was not accompanied by sustained increased pro-inflammatory cytokines with prolonged 20 w of HFD consumption. Rather, anti-inflammatory genes were upregulated while microglial sensing genes were downregulated [130]. This indicates that microglia may shift to a more anti-inflammatory or homeostatic phenotype after longer-term HFD consumption.

The regional heterogeneity of microglial responses is another important consideration. Microglia in the hypothalamic arcuate nucleus, for instance, displayed a markedly reaction to 8 weeks of HFD in a region-specific manner [130]. This underscores how microglia in different brain regions may uniquely adapted to their specific microenvironments and react differently to the metabolic challenges imposed by obesity. Furthermore, the plasticity and ability of microglia to respond to additional stimuli was preserved even after prolonged HFD feeding. When stimulated with LPS ex vivo after 8 weeks of HFD, hypothalamic microglia upregulated inflammatory genes comparable to microglia from control diet mice [130]. This indicates the microglia retain responsiveness despite adapting to the HFD conditions. Mechanically, Kim et al. [131] has been instrumental in highlighting the dynamic increase in uncoupling protein 2 (*Ucp2*) mRNA expression in the hypothalamic microglia of mice following an 8 w-HFD regimen. This increase influences mitochondrial modifications that activate microglia, further contributing to hypothalamic inflammation and the overall susceptibility to obesity. In summary, emerging evidence indicates microglial phenotypes and functions are altered in a temporal and spatial manner by obesity and HFD consumption. While often displaying pro-inflammatory features acutely, microglia may adapt with anti-inflammatory or homeostatic responses over time. Their heterogeneous phenotypes across brain regions and retained ability to respond to stimuli highlight the complexity of microglial reactions in obesity.

### Astrocytes

Astrocytes, another critical cell type in the CNS, undergo reactive astrogliosis in obesity, characterized by changes in their morphology, proliferation, and function. Similar to microglia, activated astrocytes can also secrete pro-inflammatory cytokines, further exacerbating the neuroinflammatory response. Astrocytes in 16 w-HFD consumption displayed reactive astrogliosis, characterized by altered morphology and upregulation of intermediate filaments like glial fibrillary acidic protein (GFAP) [132]. This phenotypic shift was observable early during HFD feeding, even preceding substantial weight gain. Lin et al. [133] elucidates the upregulation of disease-associated astrocyte (DAA) and microglia markers in response to an 12 w-HFD that are similar to the pathogenesis of Alzheimer’s disease. providing a direct link between dietary habits, neuroinflammation, and neurodegeneration.

The functional profile of astrocytes was also altered by obesity-induced astrogliosis. Activated astrocytes upregulate expression of pro-inflammatory cytokines, including IL-6, IL-1β, and TNF-α in mice following an 11 w-HFD regimen [134]. This creates a self-perpetuating cycle, as increased cytokine levels can further stimulate astrogliosis. Additionally, aberrant release of gliotransmitters like glutamate from reactive astrocytes can also occur, potentially impacting neuronal excitability after a 16 w of HFD [126].

Multiple signaling pathways have been implicated in driving the astrogliotic transformation of astrocytes in obesity. As Thaler et al. demonstrated, astrocyte-specific inhibition of IKKβ/NF-κB signaling mitigated weight gain, glucose intolerance, and hypothalamic inflammation induced by 11 w of HFD consumption [134]. This suggests the IKKβ/NF-κB pathway is critical for obesity-related astrogliosis and its metabolic consequences. Calcineurin signaling has also been linked to astrocyte reactivity in response to HFD for 16 w [132]. Calcineurin inhibition attenuated gliosis in the arcuate nucleus, ventromedial hypothalamus, and dorsomedial hypothalamus of HFD mice. This implicates calcineurin/NFAT as another important mediator of astrocyte activation. In summary, the morphological and functional changes accompanying astrogliosis position astrocytes as key propagators of neuroinflammatory responses in obesity models induced by high-fat feeding. Delineating the intracellular signaling pathways driving these astrocyte alterations, such as IKKβ/NF-κB and calcineurin/NFAT, will contribute to uncover therapeutic targets for mitigating obesity-associated hypothalamic inflammation.

### Monocytes/macrophages

Peripheral immune cells, particularly monocytes, have been reported to infiltrate the CNS in obesity. These monocytes can differentiate into macrophages, producing a variety of pro-inflammatory cytokines that exacerbate neuroinflammation. For instance, a bone marrow chimerism mouse model demonstrated that 15 or 30 w of HFD-induced obesity led to a 30% increase of immune cells in the CNS compared to controls [129]. Most of these cells exhibited a microglia/macrophage phenotype, being CD45^+^CD11b^+^. The ratio of CD11b^+^CD45^hi^ to CD11b^+^CD45^lo^ cells was elevated, indicating an inflammatory state. In addition to the infiltration, HFD also promotes the differentiation of monocytes into macrophages. It was demonstrated that 14 w of HFD consumption induced the Ly6c^high^ monocytes to differentiate into macrophages in the brain [135]. Another study showed that prolonged HFD consumption for 4 and 20 w led to expansion of the monocyte-derived macrophage pool in the hypothalamic arcuate nucleus, attributed to enhanced macrophage proliferation [136]. In mouse models of leptin receptor deficiency, breakdown of the BBB was found to enable macrophage infiltration into the brain of *db/db* mice [137]. Leptin resistance, glucose intolerance, and elevated cytokines like IL-1β and TNF-α accompanied the accumulation of macrophages. Notably, this study also suggested that IL-1β potentially play an important role in trafficking of peripheral monocytes into the brain. Overall, these studies indicate HFD-induced obesity facilitates the recruitment and proliferation of macrophages in the brain, propagating inflammation.

While the involvement of monocytes and macrophages in mediating neuroinflammatory responses under obese conditions is evident, it is imperative to scrutinize the methodologies employed in these studies for potential confounding factors. Specifically, investigations utilizing GFP bone marrow chimeras, such as those by Buckman et al. [129] and Baufeld et al. [130] mention above, provide critical insights into the trafficking of these immune cells in the context of obesity. However, the integral role of radiation treatment in these experimental designs warrants a careful evaluation of its impact on the observed outcomes. Radiation used to establish bone marrow chimeras, as a preparatory step for tracking immune cell migration, is known to independently alter BBB permeability and trigger pro-inflammatory responses, as detailed by Kierdorf et al. [138] This raises important considerations for interpreting studies on obesity-associated neuroinflammation: the increased permeability of the BBB and the augmented infiltration of monocytes/macrophages observed could be confounded by the radiation treatment itself, rather than being solely attributable to the effects of obesity.

Acknowledging these potential confounding effects is crucial for a comprehensive understanding of the dynamics between obesity and neurovascular integrity. Future research aimed at elucidating the specific roles of monocytes and macrophages in obesity-induced BBB disruption and brain inflammation should consider employing alternative methodologies that circumvent the need for radiation-induced bone marrow ablation. This approach will ensure a clearer delineation of the direct consequences of obesity on neuroimmune interactions and BBB integrity, free from the complicating effects of experimental interventions.

By carefully considering these methodological nuances, we can enhance our understanding of the complex interplay between obesity, BBB integrity, and the role of monocytes/macrophages in neuroinflammation, thereby paving the way for more targeted and effective therapeutic strategies.

### Pro-inflammatory factors

Obesity-induced neuroinflammation is also marked by elevated expression levels of pro-inflammatory factors. In addition to TNF-α, IL-1β, and IL-6, other pro-inflammatory molecules such as inducible nitric oxide synthase (iNOS) and cyclooxygenase-2 (COX-2) show increased expression in obesity. These molecules, produced by both infiltrating peripheral immune cells and activated resident brain cells, not only propagate inflammation but can also contribute to BBB disruption and neuronal damage. In human frontal cortex, it was reported that increased BMI has been found to cause iNOS-mediated inflammatory activity [139]. A recent finding demonstrated that iNOS promotes hypothalamic insulin resistance in obese rats. Aberrant nitrosative stress such as S-nitrosatiion (also referred to as S-nitrosylation) is closely associate with various neurological disorders such as AD and Parkinson’s Disease (PD) [140, 141]. Inhibition of central iNOS ameliorated not only glucose metabolism, but also macrophage activation induced inflammation in hypothalamus of HFD-induced obesity mice [136]. In addition, 18 or 20 w of HFD consumption also can significantly enhance the expression of COX-2 in the hippocampus of the mice [142, 143]. An increased activity of COX-2-PEG2 signaling pathway has been considered to play a key role in impairing hippocampal neuronal function and cognition [144, 145].

Additionally, the aberrant activation of the inflammasome complexes, especially NOD-like receptor thermal protein domain associated protein 3 (NLRP3), plays a vital role in obesity-induced neuroinflammatory [146]. Within the CNS, microglia accumulate lipid droplets and activate NLRP3 inflammasomes under hyperglycemic conditions due to impaired lipophagy [147]. The microglial specific inflammatory amplifier TREM1 (triggering receptor expressed on myeloid cells), resulting in the buildup of microglial TREM1, was found to aggravates the HG-induced lipophagy damage and subsequently promoted HG-induced neuroinflammatory cascades via NLRP3 (NLR family pyrin domain containing 3) inflammasome. Pharmacological blockade of TREM1 with LP17 in db/db mice and HFD/STZ mice inhibited accumulation of lipid droplets and TREM1, reduced hippocampal neuronal inflammatory damage, and consequently improved cognitive functions [147].

Together, these diverse peripheral and central inflammatory pathways contribute to BBB disruption, neurotoxicity, and cognitive deficits associated with obesity. Targeting shared processes like nitrosative stress, which interacts with iNOS signaling, may simultaneously mitigate obesity-related neuroinflammation. The upregulation of pivotal inflammatory enzymes like iNOS and COX-2 highlights the multi-faceted nature of obesity-induced inflammation and the need for strategies that address key underlying pathways.

**Fig. 4:**
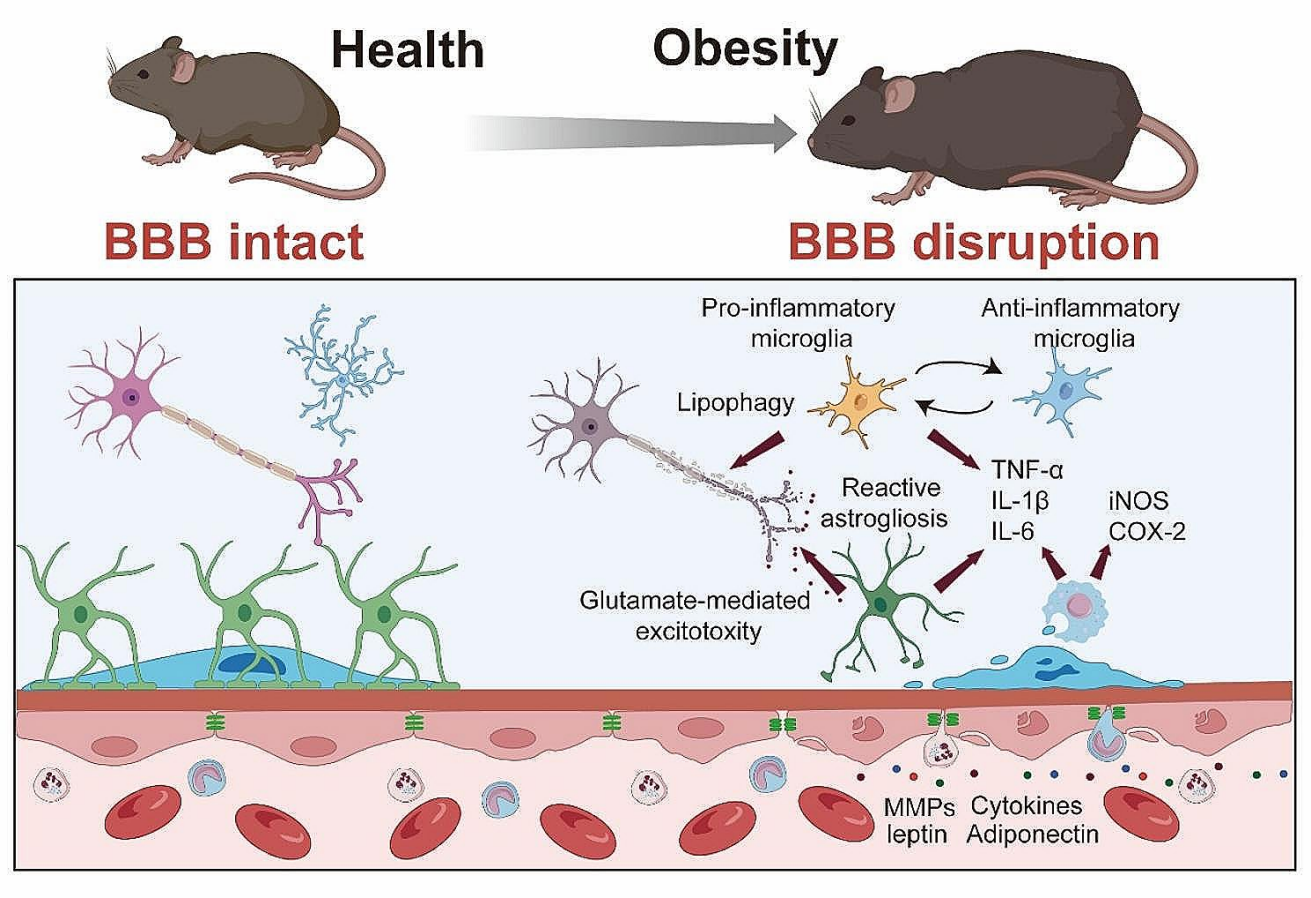
**Mechanisms of Obesity-Induced Neuroinflammation**. Enhanced microglial activation in obesity leads to increased release of pro-inflammatory cytokines (TNF-α, IL-1β, IL-6), contributing to a pro-inflammatory CNS environment. Concurrently, obesity triggers reactive astrogliosis in astrocytes characterized by morphological and functional changes, and secretion of pro-inflammatory cytokines. The infiltration of peripheral monocytes into the CNS, and differentiation into macrophages further exacerbates neuroinflammation through additional cytokine production. Elevated expression of pro-inflammatory factors such as iNOS and COX-2 in obesity are also depicted, highlighting their potential role in neuroinflammation, BBB disruption, and neuronal damage

## Dietary influences on BBB integrity and obesity outcomes

The intricate relationship between obesity and inflammation underscores the multifaceted nature of BBB dysfunction. While the systemic inflammation associated with obesity undoubtedly impacts BBB integrity, it is imperative to address the direct and indirect effects of dietary patterns on neurovascular health. The link between diet, obesity, and BBB integrity provides a unique lens through which to understand the broader implications of nutritional habits on cerebral health and disease susceptibility. Freeman et al. [148] highlights the adverse effects of a 6 month consumption of high-fat/high-cholesterol (HFHC) diet on BBB function, demonstrating a reduction in BBB integrity markers and an increase in inflammatory responses within the hippocampus in middle-aged rats. This suggests that HFHC diets, beyond contributing to obesity, may directly compromise the BBB, thereby facilitating a neuroinflammatory state that could underpin cognitive decline. Similarly, Davidson et al. [149] reported on the effects of high-energy diets on hippocampal-dependent cognitive functions and BBB integrity. Their findings emphasized that diets high in saturated fats and sugars, hallmarks of the “Western” diet, not only contribute to obesity but also impair types of learning and memory dependent on the hippocampus. The unique aspect of this research is the differentiation between diet-induced obese and diet-resistant rats, highlighting how diet affects cognitive function and BBB integrity differently based on individual susceptibility to obesity. This is particularly concerning as it implies that dietary components can directly influence cognitive functions by altering hippocampal integrity and possibly disrupting the BBB. The dietary components and direct influences on BBB was detailed in Table 1, which underscores the potential for dietary modifications as preventive or mitigative strategies against obesity-induced neurovascular alterations.

**Table 1:** Dietary patterns and their impacts on obesity development and BBB integrity

Strain	Sex	Age or Weight	Diet	Control Diet	Time	Methodology for BBB integrity	Phenotype and Mechanism of BBB dysfunction	Reference
CD-1 mice	Male	8 w	Fat: 60 kcal % Protein: 20 kcal % Carbohydrate: 20 kcal % (Research Diets, D12492)	Fat: 10 kcal % Protein: 20 kcal % Carbohydrate: 70 kcal % (Research Diets, D12450J)	16 or 36 w	Radiolabeled albumin and sucrose	Increases ^99m^Tc-albumin in the whole brain; Increases ^14^C-sucrose in hypothalamus and hippocampus; Decreases ZO-1 and Claudin-12 protein levels in whole brain lysate; Decreases ZO-1 and Occludin expression in hypothalamus.	[72]
C57BL/6J mice	Female	6 w	Fat: 40 kcal % Protein: 12.6 kcal % Carbohydrate: 47.4 kcal % (Specialty Feeds, SF07-050)	Fat: 9.4 kcal % Protein: 14.7 kcal % Carbohydrate: 75.9 kcal % (American Institute of Nutrition, AIN-93 M)	10 w	Immunofluorescence staining for IgG	Increases extravasation of IgG in cortex and hippocampus	[73]
ICR mice	Male	20–25 g	Fat: 20.1 (w/w) % Protein: 18.3 (w/w) % Carbohydrate: 51.2 (w/w) %	Fat: 5.1 (w/w) % Protein: 23.5 (w/w) % Carbohydrate: 50.3 (w/w) %	8 w	EB staining	Increases extravasation of EB in cortex; Disrupts the polymerization of ZO-1 and Occludin in brain.	[74]
C57BL/6J mice	Female	6 w	Fat: 40 kcal % Protein: 12.6 kcal % Carbohydrate: 47.4 kcal % (Specialty Feeds, SF07-050)	Fat: 9.4 kcal % Protein: 14.7 kcal % Carbohydrate: 75.9 kcal % (American Institute of Nutrition, AIN-93 M)	9 m	Immunofluorescence staining for IgG and apoB	Increases extravasation of IgG and apoB in cortex.	[75]
C57BL/6J mice	Male	12–16 w	Fat: 15.7 (w/w) % Protein: 17.6 (w/w) % Carbohydrate: 45.7 (w/w)	Fat: 4.5 (w/w) % Protein: 20 (w/w) % Carbohydrate: 52 (w/w) %	8 w	TMR-conjugated dextran and EB leakage	Increases leakage of dextran around microvessles in cortex; Increases extravasation of EB in brain. Decreases the protein levels of ZO-1, Occludin and claudin-5 in isolated brain vessels.	[77]
Sprague-Dawley albino rats	Male	60 d	Fat: 40 kcal % Protein: 21 kcal % Carbohydrate: 38 kcal % (Harlan Teklad, TD.04489)	Fat: 13 kcal % Protein: 29 kcal % Carbohydrate: 58 kcal % (Lab Diet, formula 5001)	90 d	NaFI leakage	Increases extravasation of NaFI in hippocampus; Decreases the mRNA expression of claudin-5, -12, and ZO-2 in the choroid plexus, Decreases the mRNA levels of Occludin in BBB capillaries.	[78]
C57BL/6J mice	Male	8 w	Fat: 60 kcal % Protein: 20 kcal % Carbohydrate: 20 kcal % (Research Diets, D12492)	Fat: 10 kcal % Protein: 20 kcal % Carbohydrate: 70 kcal % (Research Diets, D12450)	8 w or 12 w or 16 w	EB, NaFI and IgG leakage; TEM observation for BBB ultrastructure	Increases extravasation of NaFI in hippocampus (12 w HFD); Increases extravasation of EB in hippocampus (16 w HFD); Increases extravasation of IgG in hippocampus (12/16 w HFD); Induces involution and atrophy of tight junctions in hippocampus (16 w HFD); Reduces the coverage of pericyte in e hippocampus (16 w HFD); Increases transcytotic vesicles in endothelial cells in hippocampus (16 w HFD).	[79]
C57B6/J mice	Female	6 w	Fat: 45 kcal % Protein: 20 kcal % Carbohydrate: 35 kcal % (Research Diets, D12451)	Fat: 10 kcal % Protein: 20 kcal % Carbohydrate: 70 kcal % (Research Diets, D12450B)	until fat enough	EB and IgG leakage	Increases extravasation of EB and IgG in the ARC of neonatal offspring; Increases fenestrations in endothelial cells in the ARC of neonatal offspring; Increases the mRNA levels of claudin-1, -3 and ZO-1 in the ARC of neonatal offspring; Increases active transcellular transport in the ARC of neonatal offspring .	[82]
C57BL/6J mice	Male	7 w	Fat: 60 kcal % (SYSE, PD6001)	Fat: 10 kcal % (SYSE, D12450J)	4 m	mRNA level of TJs	Decreases the mRNA levels of ZO-1 and Occludin in hippocampus and prefrontal cortex.	[83]
C57BL/6J mice	Male	6 w	Fat: 45 kcal % (Research Diets)	Fat: 17 kcal % (Mucedola Srl)	19 w	Albumin leakage	Increases the number of scattered albumin-positive neurons (extravasated albumin is taken up by neuronal cells) in hippocampus; Decreases the transcription of tight junction protein 1, Occludin and Claudin5.	[84]
C57BL/6J mice	Male	2 m	Fat: 45 kcal % Protein: 20 kcal % Carbohydrate: 35 kcal % (D12451)	Fat: 13.5 kcal % Protein: 28.5 kcal % Carbohydrate: 58 kcal % (Purina 5001)	2 m	Comparative proteomic	Downregulates 47 proteins in the cerebral microvessels, including cytoskeletal proteins, chaperons, enzymes, transport-related proteins, and regulators for transcriptional and translational activities; Upregulates two proteins, involved in mRNA transport and processing.	[76]
C57BL6/J mice	Male	6 w	Fat: 29.9 (w/w) % Protein: 13.6 (w/w) % Crude Fibre: 4.7 (w/w) % (Specialty Feeds, SF14-088)	Fat: 4 (w/w) % Protein: 13.6 (w/w) % Crude Fibre: 4.7 (w/w) % (Specialty Feeds, AIN-93 M)	4 or 24 w	IgG leakage	Increases extravasation of IgG in cortex and hippocampus.	[125]
Sprague-Dawley albino rats	Male	250–275 g	Fat: 40 kcal % Protein: 21 kcal % Carbohydrate: 38 kcal % (Harlan Teklad, TD.04489)	Fat: 13 kcal % Protein: 29 kcal % Carbohydrate: 58 kcal % (Lab Diet, formula 5001)	until fat enough	NaFI leakage	Increases extravasation of NaFI in hippocampus and cortex.	[149]
Fischer rats	Female	6 m	Hydrogenated coconut oil: 10 (w/w) % Cholesterol: 2 (w/w) % (MP Biomedicals, ‘Custom Diet D2-AIN93 without choline bitartrate and with 2% cholesterol’)	Fat: 13 kcal% Protein: 34 kcal% Carbohydrate: 53 kcal% (Harlan Teklad, 8656)	6 m	SMI-71 (an antibody specific to the rat endothelial-barrier protein)	Decreases the immunoreactivity of SMI-71in cortex and hippocampus; Increases the protein levels of Occludin in hippocampus.	[148]
C57BL/6 N mice	Male	8 w	Fat: 60 kcal % Protein: 19 kcal % Carbohydrate: 21 kcal % (Ssniff, D12492) (for acute HFD)	Fat: 9 kcal % Protein: 34 kcal % Carbohydrate: 57 kcal % (Ssniff, V1554)	3 d-4 w	mRNA levels of transporters	Decreases the mRNA levels of Glut-1 after 3 d HFD, until 1 w HFD. Decreases the immunoreactivity of Glut-1 after 3–7 d HFD. The expression of Glut-1 is restored after 4 w HFD and the serum levels of VEGF increases.	[95]
C57BL/6 mice	Male	8 w	Fat: 59 kcal % Protein: 18 kcal % Carbohydrate: 23 kcal % (Test Diet, 58G9)	Fat: 12 kcal % Protein: 18 kcal % Carbohydrate: 70 kcal % (Test Diet, 58G7)	3 d	Protein levels of transporters	Decreases the expression and function of Glut-1.	[96]
C57BL/6 N mice	Male	8 w	Fat: 56.7 kcal % Protein: 20 kcal % Carbohydrate: 23.1 kcal % (CLEA, HFD32)	Fat: 12 kcal % Protein: 29 kcal % Carbohydrate: 59 kcal % (CLEA, CE-2)	2 or 10 w	EB leakage; Transporter and receptor expression analyze	2 w HFD: Does not induce the extravasation of EB; Decreases the expression of Claudin-5, Occludin and ZO-1; Decreases the expression of Mdra1, Glut-1, P-gp, LAT1, Mct1, TfR and Lrp1 in the brain capillary; 10 w HFD: Increases the expression of Mdra1, Glut-1, P-gp, LAT1, Mct1 and TfR in the brain capillary; Increases the expression of Claudin-5, Occludin and ZO-1.	[97]
Mongrel dog	Male	27–40 kg	Fat: 80 kcal % (Harlan Teklad)	Fat: 17 kcal % (Harlan Teklad)	7 w	Marked intravenous insulin injection	Decreases the CNS insulin level.	[100]
CD-1 mice	Male	8–10 w or 56–64 w	Not mentioned	Not mentioned	Not mentioned	Intravenous radiolabeled insulin injection; Transcardiac radiolabeled insulin brain perfusion	Decreases the transport of insulin across the BBB; Regulates the BBB transport by circulating factors such as the triglyceride triolein (increases the brain uptake of insulin).	[101]
Sprague Dawley rats	Male	8 w	Fat: 60 kcal % Protein: 20 kcal % Carbohydrate: 20 kcal % (Research Diets, D12492)	Normal chow diet (No details)	4 w	Radiolabeled insulin uptake and degradation tests	Decreases isolated-BECs insulin uptake; Increases NF-κB binding activity.	[102]
C57BL/6NHsd mice	Male	6 w	Fat: 40 kcal % Protein: 16 kcal % Carbohydrate: 44 kcal % (Test Diet, 5TJN) Sugar: 42 g/L, add to the water	Fat: 18 kcal % Protein: 24 kcal % Carbohydrate: 58 kcal % (Harlan Teklad, 2018)	14 w	Intraperitoneal glucose injection; Glucose and insulin tolerance tests	Reduces brain insulin resistance by decreasing tyrosine phosphorylation of insulin receptor and increasing serine phosphorylation of IRS-1; Induces inflammatory (NF-κB, JNK) and stress responses (p38 MAPK, CHOP).	[104]
Lean FA/FA rats or genetically obese Zucker fa/fa rats	Male	9 w	For lean FA/FA rats: Fat: 40 (w/w) % (Harlan Teklad, TD-97,113)	For lean FA/FA rats: Fat: 4.50 (w/w) % (Lab Diet, 5001)	For lean FA/FA rats: 10 w	Intravenous radiolabeled leptin injection	Decreases the permeability of leptin at the BBB; Induces the saturation of leptin receptor at the BBB.	[108]
Suffolk × Greyface crossbreeds sheep	Male	1 y	Comprising 50% chopped hay, 30% rolled barley and 9% soyabean meal, etc.: *ad libitum* for 40 w; then restricted to 0.75 × maintenance for 16 w	Comprising 50% chopped hay, 30% rolled barley and 9% soyabean meal, etc.: restricted to 1.1 × maintenance for 40 w; then *ad libitum* for 16w	56 w	*i.c.v.* injection of marked leptin	Decreases the transport of leptin across the BBB.	[109]
C57BL/6 and AKR mice	Male	4 w	Fat: 45 kcal % Protein: 20 kcal % Carbohydrate: 35 kcal % (Research Diets, D12451)	Fat: 10 kcal % Protein: 20 kcal % Carbohydrate: 70 kcal % (Research Diets, D12450)	56d	Intra-cerebroventricular and peripheral leptin injection	Induces resistance to peripherally administered leptin, while retaining sensitivity to centrally administered leptin.	[111]

## Emerging therapeutic strategies in tackling obesity-induced BBB dysfunction

In addressing the complexities of obesity-induced metabolic syndrome and its impact on the central nervous system, a spectrum of experimental therapies is currently under investigation, each targeting distinct pathological mechanisms. One promising approach involves the decrease of BBB permeability. Agents such as Palmitoylethanolamide, Topiramate, and Nicotine have demonstrated potential in modulating BBB dynamics, suggesting a pivotal role in mitigating obesity-related neurological sequelae in mice subjected to 10–36 w of HFD or 10 w of high saturated fatty acids diet consumption [72, 73, 84]. Among these, Palmitoylethanolamide has shown promise, not only in attenuating anxiety-like behavior but also in modulating neurotransmitter levels such as dopamine turnover and γ-aminobutyric acid (GABA) levels in the amygdala from mice subjected to 19 w of HFD consumption [84]. Additionally, it has been found to reduce systemic inflammation markers like TNF-α and IL-1β, attenuate hypothalamic injury, and decrease neuroinflammation and BBB permeability in the hippocampus. However, while its effects on BBB permeability are notable, further investigation is necessary to fully understand its long-term efficacy and safety profile, particularly in relation to chronic administration and potential systemic effects.

Topiramate, another therapeutic agent, has been demonstrated its capability to decrease BBB permeability in mice subjected to 13 and 36 w of HFD [72]. It achieves this through increasing the expression of tight junction proteins like ZO-1 and Claudin-12, which are crucial in maintaining BBB integrity. Topiramate also exhibits properties that inhibit oxidative stress, a common pathological feature in obesity-related neurological disorders. In this regard, agents like Dapsone and Resveratrol have garnered attention for their potential to protect tight junctions and reduce BBB disruption in mice following 8 w of HFD [74, 77]. Dapsone, for instance, has been found to decrease brain microvascular leakage, which is often exacerbated in obesity. It does so by inhibiting the oxidation of low-density lipoproteins (LDL), a process that is detrimental to the BBB [77]. Additionally, Dapsone has shown efficacy in protecting tight junction proteins such as ZO-1, Claudin-5, and Occludin, further enhancing its role in preserving BBB integrity in mice following 8 w of HFD. Similarly, Resveratrol, a naturally occurring polyphenolic compound, has been demonstrated its capacity to fortify BBB tight junctions. Its neuroprotective properties extend beyond just maintaining the BBB; Resveratrol also exhibits antioxidative and anti-inflammatory effects, which are beneficial in addressing the multifactorial aspects of obesity-induced neural damage in mice subjected 8 w of HFD [74]. However, while these agents show promise, their clinical application faces challenges. The variability in individual responses and potential side effects, such as hypersensitivity reactions with Dapsone, require careful consideration. Future research must focus on optimizing these therapies, possibly through targeted delivery systems or combination therapies, to enhance their protective effects on the BBB while minimizing adverse reactions. Despite these benefits, the challenge with Topiramate lies in its potential side effects, such as cognitive disturbances and weight loss, which might limit its use in certain patient populations. Future research should focus on optimizing its dosage and delivery mechanisms to maximize its therapeutic benefits while minimizing adverse effects. Besides the aforementioned strategies, other pharmacological effects pivotal in managing obesity-induced complications include the modulation of oxidative stress and cellular death, the regulation of metabolic pathways, and the enhancement of neural regeneration, each contributing uniquely towards mitigating the multifaceted challenges posed by obesity.

In conclusion, the emerging therapeutic strategies discussed in this section underscore the complexity and multidimensionality of tackling obesity-induced metabolic syndrome and its neurological implications. From enhancing BBB integrity to addressing oxidative stress, metabolic dysregulation, and promoting neuroregeneration, each approach offers a unique angle in combating the extensive impact of obesity [150, 151]. This multifaceted approach not only broadens our understanding but also paves the way for innovative and comprehensive treatments in the ongoing battle against obesity-related neurological disorders. The major experimental therapies are summarized in Table 2, which presents a curated list of compounds demonstrated to exert protective effects against the adverse consequences of obesity on the brain, particularly focusing on maintaining or restoring BBB integrity. Each compound included has been selected based on empirical evidence from studies highlighting its efficacy in counteracting obesity-induced neurovascular dysfunction.

**Table 2:** Emerging therapeutic strategies for alleviation of obesity-related BBB dysfunction

Therapeutic strategies	Protective effects and mechanisms in obesity-induced BBB dysfunction	References
**Palmitoylethanolamide**	Decreases BBB permeability; Restores tight junction transcription. Attenuates hypothalamic injury; Reduces the systemic inflammation (TNF-α, IL-1β, etc.).	[84]
**Adora2a antagonism**	Decreases BBB permeability.	[79]
**Topiramate**	Decreases BBB permeability; Increases expression of TJs (ZO-1, Claudin-12); Inhibits oxidative stress.	[72]
**Dapsone**	Decreases brain microvascular leakage; Protects TJs (ZO-1, Claudin-5, Occludin); Reduces lysosome accumulation in cerebral microvessels.	[77]
**Nicotine**	Decreases BBB permeability.	[73]
**Resveratrol**	Protects against breakage of the BBB; Reduces the disruption of polymerization of TJs (ZO-1, Occludin).	[74]
**Nutraceutical agents**	Decreases the BBB damaging effect; Reduces oxidative stress and neurovascular inflammation.	[75]

## Conclusion and future directions

The intricate association between obesity and the BBB has been the primary focus of this review. We have delved into the fundamental understanding of BBB dysfunction in the context of obesity, detailing the altered BBB permeability, transport dysfunction, and neuroinflammation, each contributing to a multifaceted pathophysiological landscape that paves the way for obesity-related neurological disorders. Despite the progress made, several gaps persist in our understanding of obesity-induced BBB dysfunction. For instance, more in-depth studies on the temporal relationship between obesity and BBB dysfunction could provide insight into the initial triggers of these changes. Furthermore, it remains unclear whether BBB dysfunction is a universal feature of obesity or if it varies with factors such as the degree and duration of obesity, age, sex, and genetic predisposition. Moreover, the potential reversibility of obesity-induced BBB changes and the optimal strategies for achieving such reversibility warrant exploration.

Recognizing BBB dysfunction’s role in obesity-related neurological diseases holds significant promise for future therapeutic advancements. By better understanding the interactions between BBB dysfunction and neurodegeneration, we may discover new strategies to mitigate or even prevent the deleterious effects of obesity on the CNS. There lies immense potential in targeting the BBB for therapeutic intervention, with the promise of not only alleviating obesity-induced BBB dysfunction but also mitigating its downstream effects, including neuroinflammation and neurodegeneration.

In conclusion, obesity-induced BBB dysfunction represents an area of research with implications extending beyond the realm of obesity to a broad spectrum of neurological disorders. A more comprehensive understanding of obesity’s influence on the CNS would ultimately benefit those affected by obesity and its neurological consequences.

## Data Availability

No datasets were generated or analysed during the current study.
